# Participant Perspectives on Community Qigong for People with Multiple Sclerosis

**DOI:** 10.1089/imr.2022.0079

**Published:** 2023-02-01

**Authors:** Lita Buttolph, Lindsey Wooliscroft, Ryan Bradley, Heather Zwickey

**Affiliations:** ^1^ Helfgott Research Institute, National University of Natural Medicine, Portland, OR, USA.; ^2^ Department of Neurology, Oregon Health and Science University, Portland, OR, USA.; ^3^ Department of Neurology, VA Portland Health Care System, Portland, OR, USA.

**Keywords:** multiple sclerosis, pragmatic design, qigong, qualitative research

## Abstract

**Introduction:**

Multiple sclerosis (MS) is a progressive neurodegenerative disorder affecting motor and nonmotor function including physical and cognitive decline, fatigue, anxiety, and depression. Qigong is a mind–body self-care practice with the potential to address MS symptoms. Publicly available community qigong classes may provide opportunities for people with MS to access qigong, but little is known about the risks and benefits. A mixed methods study of community qigong was conducted for people with MS. In this article, the results of this qualitative analysis to identify benefits and challenges faced by people with MS attending community qigong classes were presented.

**Methods:**

Qualitative data were collected from an exit survey of 14 study participants with MS who enrolled in a pragmatic trial of community qigong classes for 10 weeks. Participants were new to community-based classes offered but some had experience with qigong/tai chi/other martial arts or yoga. Data were analyzed using reflexive thematic analysis.

**Results and Discussion:**

Seven common themes were identified from this analysis: (1) physical function, (2) motivation/energy, (3) learning, (4) dedicating time for self, (5) meditation/centering/focus, (6) relaxation/stress relief, and (7) psychological/psychosocial. These themes reflected both positive and negative experiences with community qigong classes and home practice. Self-reported benefits centered around improved flexibility, endurance, energy, and focus; stress relief; and psychological/psychosocial benefits. Challenges included physical discomfort including short-term pain, balance difficulty, and heat intolerance.

**Conclusion:**

The qualitative findings provide evidence to support qigong as a self-care practice that may benefit people with MS. The challenges identified in the study will help to inform future clinical trials of qigong for MS.

**Trial Registration:**

ClinicalTrials.gov (CTR#: NCT04585659).

## Introduction

Multiple sclerosis (MS) is an autoimmune disorder of the central nervous system affecting ∼1 million people in the United States.^1,2^ Neuronal demyelination associated with MS can lead to a variety of motor and nonmotor symptoms including muscle weakness, pain, numbness, and spasticity, as well as cognitive impairment, fatigue, anxiety, and depression. The individual economic burden of MS is estimated to be $4.8 million over a lifetime.^3^ Although disease-modifying therapies can help slow disease progression, symptom management often involves a combination of behavior and lifestyle changes, environmental and physical modifications, rehabilitation, and medical interventions.^4^ Finding cost-effective therapies to help address the complex symptom presentations commonly experienced by people with MS is critical.

Qigong is an ancient Chinese movement art, similar to modern medical versions of tai chi,^5^ developed to promote self-healing and longevity through mindful movement, breathwork, visualization, and meditation. Evidence suggests qigong can improve physical strength, flexibility, balance, neurointegration, emotional regulation, self-efficacy, and proprioceptive and interoceptive awareness.^6–25^ Clinical trials of other neurologic disorders, such as Parkinson's disease and fibromyalgia, suggest that qigong can improve both motor and nonmotor symptoms, supporting its potential benefit for people with MS.^12,13,16,18,20,21,23,24,26–34^

There have been a limited number of small studies of qigong and tai chi for people with MS that have found improvements in quality of life, functional balance, increased flexibility, leg strength, gait and reduced pain, fatigue, and depression.^35,36^

Accessing qigong classes in the real world can pose different challenges as well as provide opportunities not observed in more controlled clinical trial settings. A mixed methods randomized control pragmatic feasibility trial of community qigong for people with MS was conducted.^37^ As part of the study, qualitative data were collected to capture participant perspectives about the qigong intervention.

Qualitative analyses have been used in several qigong and tai chi studies to corroborate quantitative findings, generate new hypotheses, and develop a more nuanced understanding of how these mindful movement therapies affect different populations.^38–43^ This article presents qualitative findings to better understand perceived benefits and challenges of community qigong for people with MS.

## Materials and Methods

### Study design and recruitment

In total 20 adults, 18 years and older, were recruited from the Portland Metropolitan Area with a self-reported diagnosis of any type of MS, ability to walk 50 feet (15.24 m) without an assistive device, and stable on disease-modifying medications or medication for balance 3 months before baseline. Candidates who were pregnant or nursing; participated in a regular qigong, tai chi, or yoga within 6 months before baseline; or had an MS relapse within 30 days before baseline were excluded.

More details about this recruitment strategy can be found elsewhere.^37^ The study was conducted from January 2017 to March 2018. The study protocol was approved by the National University of Natural Medicine institutional review board and registered with ClinicalTrials.gov (CTR#: NCT04585659).

### Study intervention

This study utilized a pragmatic design in which participants were randomized to weekly pre-existing public qigong classes for 10 weeks or a wait-list control. Participants randomized to the qigong group (QG) attended 60–90 min prevetted qigong classes, and were encouraged to practice at home for at least 10 min per day. Class attendance was documented by the class instructor and participant through an attendance card. Participants documented their home practice using a home practice log.

The wait-list control group (CG) was asked to refrain from qigong, tai chi, or yoga during the 10-week intervention period but was encouraged to continue with pretrial exercise and other self-care practices. After the 10-week period, CG was invited to participate in 10 weeks of qigong. Outcome measures at baseline, week 11, and, for the CG who opted to do 10 weeks of qigong, week 22 were collected.

Qigong instructors were selected based on the following criteria: a minimum of 5 years of teaching experience, experience teaching people with limited mobility, teach weekly qigong classes open to any level, and allow modified and/or seated options. Further details regarding the methods are available in a prior publication.^37^

### Outcome measures

Both quantitative and qualitative outcome measures were collected in this study. Quantitative results are reported elsewhere.^37^ To better understand how participants responded to the qigong intervention, qualitative data were collected about participants' experience with the study through an exit survey administered within 1 week of their last qigong class. Table 1 provides a list of the qualitative questions that were analyzed from the exit survey. The survey was administered using the Research Electronic Data Capture (REDCap^®^) electronic data management system.

**Table 1.** Qualitative Questions from the Exit Survey

1	Did you experience any discomfort (physical or emotional) related to this study? (If so, please explain)
2	Please use this space to describe any benefits you may have experienced from the qigong classes
3	What obstacles did you face while attending qigong classes? (Other)
4	Please describe any benefits you experienced from practicing at home
5	Please describe any challenges you had practicing at home

### Data analysis

Qualitative data were analyzed using reflexive thematic analysis.^44^ One member of the research team (L.B.) reviewed and coded the raw data to identify common themes for each question. A second member of the team (L.W.) separately assigned themes identified by the first reviewer to each response. The two researchers then reviewed the responses and themes together to further reflect on the themes, discuss discrepancies, and clarify interpretations. Bryne describes this process as achieving “richer interpretations of meaning, rather than attempting to achieve consensus of meaning.”^44^ Then a second round of analysis was conducted to further consolidate the themes.

## Results

Table 2 provides baseline characteristics of study participants who completed the qualitative portion of the exit survey (*n* = 14); 1 participant completed the quantitative portion but not the qualitative portion and is not included in the demographic table. Most participants were white college-educated females with relapsing–remitting MS. About half of the participants from each group were taking disease-modifying therapies (medications) for their MS and reported none-to-moderate disability levels. The QG reported more moderate disability (86%) than the CG (29%). Most participants engaged in moderate exercise at least once per week before joining the study, but the CG reported more vigorous weekly exercise (71%) than the QG (43%).

**Table 2.** Baseline Demographics of Study Participants Who Completed the Exit Survey

	QG (***n*** = 7)	CG (***n*** = 7)
Mean age in years (SD)	45 (8.8)	47 (12.7)
Sex, %		
Female	57	71
Education, %		
College degree or more	86	71
Race/ethnicity, %		
White	86	100
Hispanic/Latino	14	0
Type of MS, %		
Relapsing remitting	86	100
Secondary progressive	14	0
Mean years since MS diagnosis (SD)	13.4 (6.6)	7.4 (6.6)
Currently taking DMTs,^a^ %	43	57
Severity of MS disability,^b^ %		
None^c^	14	14
Mild^d^	0	57
Moderate^e^	86	29
Activity level, %		
Vigorous exercise ≥weekly^f^	43	71
Moderate exercise ≥weekly^g^	86	100
Experience with qigong, tai chi, other martial arts, or yoga	57	71

^a^ Disease-modifying therapies included Rebif (1), Copaxone (1), Tecfidera (2), Tysabri (1), other (2).

^b^ Modified from the EDSS.^57^

^c^ No or minimal MS symptoms, no limitations in walking ability or daily activities.

^d^ Noticeable MS symptoms but no limitations in walking ability or daily activities. Able to walk a ≥1 block without support.

^e^ Many MS symptoms affecting daily activities but able to walk ≥1 block without support.

^f^ Vigorous-intensity physical activity that makes you sweat or puff and pant ≥20 min per day (e.g., heavy lifting, digging, jogging, aerobics, and fast bicycling).

^g^ Moderate-intensity physical activity or walking that increases your heart rate or makes you breathe harder than normal for ≥30 min per day (e.g., brisk walking, carrying light loads, and bicycling at a regular pace).

CG, control group; EDSS, Expanded Disability Status Scale; MS, multiple sclerosis; QG, qigong group; SD, standard deviation.

Participants included those who completed the 10-week qigong intervention and those who discontinued. Table 3 provides a breakdown of exit survey completers, based on group allocation. Of the 14 participants who completed the qualitative portion of the exit survey, 7 were from the QG, of whom 1 discontinued; and of 7 from the CG who opted to join the qigong intervention during the cross-over phase, 4 discontinued.

**Table 3.** Participants from Qigong Group and Control Group Who Provided Qualitative Data from Exit Survey

	Total	QG	CG
Provided qualitative data from exit survey	14	7	7
Completed 10 weeks of qigong	9	6	3
Discontinued qigong intervention	5	1	4

A total of seven common themes were identified using the reflexive thematic analysis: (1) physical function, (2) motivation/energy, (3) learning, (4) dedicating time for self, (5) meditation/centering/focus, (6) relaxation/stress relief, and (7) psychological/psychosocial. Responses often included a combination of themes, and themes could reflect both positive and negative experiences.

### Self-reported benefits from qigong

Table 4 lists themes with exemplary participant responses for benefits reported from qigong class and home practice. Reported improvements in physical function centered around increased flexibility and endurance. Other benefits reported were improved energy after class, improved mood and focus, stress reduction, learning new information, and having dedicated time for self-care.

**Table 4.** Themes Generated from the Reflexive Thematic Analysis with Sample Participant Responses Regarding Benefits from Qigong Class and Home Practice

Theme	Sample of participant responses
Physical function	• “Physically, I'm able to stand, walk, run, carry things, in fact, do all physical activities better and longer without trouble or needing help.” P12 • “Increased functionality and control of one of my legs, majority of the help was just from the stretching.” P10 • ”Increased flexibility.” P5 • “Learning self-massage techniques has been helpful to alleviate stiffness in my hands.” P17
Motivation/energy	• “I felt I had slightly more energy immediately following class. My fatigue seemed to return to baseline, however, about 45–60 minutes after.” P2 • “Improved energy and mood after classes.” P5
Learning	• “Learned more about qi flows and meridians and how to help myself using them” P1
Dedicating time for self	• “Finding time for me…” P1
Meditation/centering/focus	• “Gave me time to practice meditation and benefit from others in the class and their energy.” P1 • “[I] was able to practice self-massage and balancing on one foot. This was helpful in focusing my attention to what is going on with my body.” P17
Relaxation/stress relief	• “Symptom reduction. Stress relief.” P3 • “Felt relaxing.” P8 • “I felt like I slept better and moved more.” P13
Psychological/psychosocial	• “My negative inner voices have been lessened, I'm noticeably less depressed and less dragged down by mental issues…” P12 • “I felt more centered emotionally and I felt energized.” P11

P[#]: Indicates participant identification number.

Of the 14 people who completed the exit survey, 6 reported some benefit from qigong classes in physical function, including increased activity, endurance, and benefits from stretching. Four participants reported psychological improvements including overall mood and depression. Four people reported improved motivation/energy. Three participants mentioned learning new information, including theory and techniques. Two people mentioned improved relaxation, and time to meditate and focus. One person mentioned dedicating time for self.

For benefits from home practice, four participants reported relaxation/stress relief from home practice. Three participants reported improved physical function. Time for meditation/centering/focus was mentioned by two participants. One participant reported dedicating time for self, and another reported improved motivation/energy.

### Discomfort/challenges with qigong

Of the 14 people who completed the exit survey, 4 reported some physical discomfort with qigong. Table 5 provides responses to questions about discomfort and obstacles related to the qigong intervention, which center around physical discomfort. One participant described challenges with being socially and physically uncomfortable in a class due to the room temperature, not enough support from the instructor, and class composition (e.g., others who did not have MS). This was categorized under both “learning” (related to the learning environment) and “meditation/centering/focus,” (related to inability to feel centered).

**Table 5.** Participant-Reported Discomfort/Challenges with Qigong Classes

Theme	Participant responses
Physical function	• “Some bending and stretching poses were strenuous and mildly uncomfortable.” P5
	• “About the 4th week my legs started to get worse. Extreme spasticity.” P8
	• “Balance issues related to nerve induction speed discrepancies were too distracting to effectively perform the internal work required for full effect, only solution I could think of is doing the exercises in a pool to lessen the effect of gravity.” P10
	• “Some back and leg nerve pain until I modified two of the exercises.” P11
Learning and meditation/centering/focus	• “The one…class I attended was physically and emotionally uncomfortable—I didn't feel like the instructor gave enough explanation of modification of poses, the room was hot, and I was uncomfortable with many others in class without MS and who were more advanced in qigong.” P5

P[#]: Indicates participant identification number.

Table 6 lists participants' discomfort/challenges with home practice. Lack of motivation/energy was as common theme mentioned by participants, followed by lack of time (categorized under “dedicating time for self”), and difficulty remembering movements (categorized under “learning”). Inability to focus, feeling hot, and feeling self-conscious about practicing at home were other barriers mentioned. The lack of social support from the class also hindered home practice.

**Table 6.** Participant-Reported Discomfort/Challenges with Qigong Home Practice

Theme	Sample participant responses
Motivation/energy	• “It was very difficult to motivate myself to practice when I was experiencing intense fatigue.” P2
	• “Motivation…” P3
	• “Fatigue kept me from doing daily practice.” P17
	• “Discipline to do it.” P13 • “Time and energy.” P8 • “Got warm, hot and fatigued…” P5
Learning	• “Couldn't remember poses or warm-ups from class. I needed printed handout of warm up exercises to do on my own at home.” P5 • “…probably not doing many moves as instructed.” P9
Dedicating time for self	• “Finding time to do it.” P1 • “I did not have time to do as much as I would have liked to do each day and found that frustrating. Often, I ended up doing just the 10 minutes.” P11
Meditation/energy/focus	• “Focus. More distractions…” P9
Psychological/psychosocial	• “It was much easier to practice with the guidance of the class.” P3 • “Having the class there is a great motivation to continue when you become physically (or mentally) fatigued.” P12

P[#]: Indicates participant identification number.

## Discussion

The results of this qualitative analysis provide preliminary data regarding individual benefits and challenges experienced by participants with mild-to-moderate MS symptoms in community qigong classes. These self-reported benefits can be summarized around themes related to physical function, energy, focus, stress relief, and psychological/psychosocial benefits. Reported benefits of qigong home practice included dedicating time for relaxation, meditation, and centering as well as improvements in physical function and energy.

This distinction between group classes and home practice is noteworthy, and each has distinct benefits and challenges. For example, home practice may be more stress reducing by eliminating travel needs and offering the safety and privacy of being at home. Motivation to practice at home may be a challenge, however, while group practice helps with accountability and offers social support and immediate feedback from instructors. Exercise adherence studies have found similar findings.^45^

It is important to note that self-reported physical benefits were not captured in this quantitative analysis of validated outcome measures, although some quantitative within-group improvements in mental health and fatigue were observed after 10 weeks of qigong.^37^ This discrepancy could be explained by reporting bias, or other factors such as the lack of sensitivity of the quantitative outcome measures in capturing effects. Another possibility is that participants experienced improvements immediately after qigong class but these were not sustained over time. This raises the question of whether and what specific dosing of qigong could achieve longer term benefits.

Challenges reported by participants included physical discomfort including some short-term pain, balance difficulty, and heat intolerance. One participant who discontinued daily stretching upon beginning the trial had a worsening of leg spasticity. Some participants expressed emotional discomfort in being in a class with people who did not have MS and were more advanced in qigong. Difficulty with home practice centered around motivation, fatigue, and the time commitment. The ability to recall movements from class was also challenging.

Pragmatic studies test the effectiveness of an intervention in a real-world setting to maximize applicability and generalizability.^46^ The National Institutes of Health recognizes the need to promote health research that can be readily applied to clinical settings, is cost-effective, and allows for efficiency in data collection, dissemination, and implementation.^47^ This study is unique in that it describes qualitatively the benefits and challenges of qigong in a real-world setting for people with MS.

A previous pragmatic design of tai chi for osteopenia assessed qualitatively opportunities and barriers around recruitment and adherence.^43^ Qualitative analyses of qigong and tai chi in other conditions (e.g., chronic obstructive pulmonary disease, poststroke recovery, heart attack, cancer, and low-back pain) found improvements in cognitive and behavior changes that promoted greater physical and psychological function.^38,42,48–50^ The small number of qigong or tai chi studies in MS report quantitative outcome measures but lack qualitative information regarding specific benefits and challenges faced by study participants.^51^

Future studies might consider more meditative styles of qigong for people with MS, as suggested by Mills, focused on developing body awareness and alignment.^52^ Mills suggests a “water approach” style of qigong that encourages “softening and flowing” versus a “fire approach” that involves more forceful breathing and vigorous movements, which may exacerbate symptoms of heat intolerance commonly found with MS.^52^

Awareness of the class environment, such as presence of air conditioning, and chairs or other equipment to help with balance, might be an important consideration for people with MS when investigating qigong classes in the community. MS-specific classes are another consideration. Written or video instructions may also be helpful to support home practice. These considerations may be broadened to inform other physiotherapy interventions in MS.

One way to understand how qigong may benefit people with MS is to look more closely at its various components: stretching, self-massage, attention to body position, muscle strengthening, rhythmic movements, visualization, attention to breath, and meditation. Each of these components may contribute to the qualitative benefits reported in this study. One possible mechanism of qigong is through development of adaptive body awareness.^53^ Colgan et al have defined adaptive body awareness as the “capacity to move from thinking about physical symptoms (e.g., interpreting, appraising, ruminating with fearful, hypervigilance) to a state of perceptual attentive presence within the body (p.4).”^53^

**FIG. 1.**
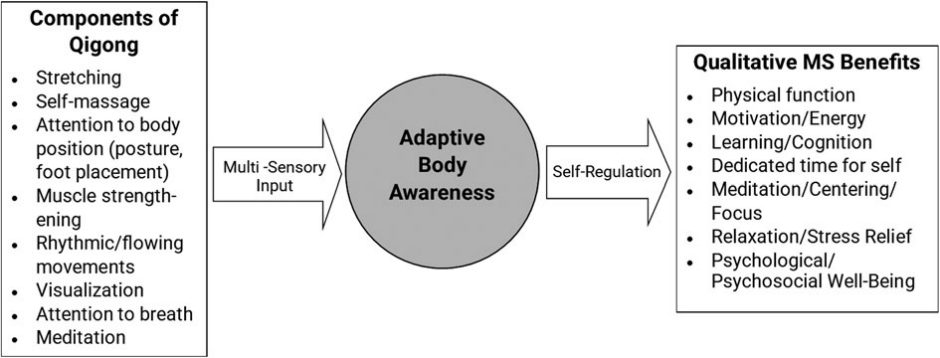
Possible mechanisms of qigong through adaptive body awareness on qualitative MS benefits. MS, multiple sclerosis.

Through the integration of intentional movement and meditative state of mind, qigong may stimulate adaptive multisensory neuropathways that promote homeostasis and well-being. Future research could detail the connection between qigong, multisensory awareness, self-regulation, and observed health benefits (Fig. 1).

Limitations of this study include small sample size and limited qualitative data. Because qualitative data were collected through survey, there was no opportunity to ask for clarification or elaboration as is possible in an interview or focus group. Thus, there is the possibility of misinterpretation of responses. Responses may be subject to reporting bias by asking about benefits and challenges. Since exit survey data were collected at the end of the trial, there is also the potential for recall bias. This study was limited geographically to the Portland Metropolitan Area, and qigong instructors were also vetted. Participants' experiences may not be reflective of other areas of the country or other qigong styles and instructors. Other qigong instructors may have different levels of experience working with people with MS and may vary in their ability to provide modifications to movements.

One of the advantages of this analysis is that data from participants who dropped out of the qigong intervention were captured. Understanding qualitatively the reasons for discontinuing a study can help shed light on what works and does not work for people with MS and improve retention in future studies. These qualitative data were also valuable in providing a context for understanding some of the quantitative results.

## Conclusion

Qualitative findings support community qigong as a feasible self-care practice for people with MS with potential benefits to physical function, energy, focus, stress relief, and mood. Potential challenges may include short-term pain, heat intolerance, balance issues, and lack of motivation and fatigue with home practice. Developing a qualitative understanding of the benefits and challenges of community qigong classes for people with MS provides a contextual framework for interpreting quantitative outcomes and will help to inform future clinical trials.

## Abbreviations Used

CG: control group
EDSS: Expanded Disability Status Scale
MS: multiple sclerosis
QG: qigong group
SD: standard deviation
